# The Effect of Low-Frequency Magnetic Fields with Low Induction and Red LED Light on Keratinocyte Biological Activity—An In Vitro Research Model

**DOI:** 10.3390/ijms252212099

**Published:** 2024-11-11

**Authors:** Renata Woźniacka, Agnieszka Lechowska-Liszka, Beata Stenka, Aneta Bac, Joanna Homa, Magdalena Chadzińska, Anna Ścisłowska-Czarnecka

**Affiliations:** 1 Institute of Applied Sciences, Academy of Physical Education, 31-001 Cracow, Poland; renata.wozniacka@awf.krakow.pl (R.W.); agnieszka.liszka@awf.krakow.pl (A.L.-L.); aneta.bac@awf.krakow.pl (A.B.); 2 Department of Applied Cosmetology, University of Physical Education and Sport, 80-309 Gdansk, Poland; beata.stenka@awf.gda.pl; 3 Institute of Zoology and Biomedical Research, Jagiellonian University, 31-001 Cracow, Poland; joanna.homa@uj.edu.pl (J.H.); magdalena.chadzinska@uj.edu.pl (M.C.)

**Keywords:** keratinocyte, magnetostimulation, LED therapy, magneto-LED therapy, wound healing

## Abstract

For several decades, there has been growing interest in the influence of low-frequency magnetic fields (LFMFs) and red LED light on the healing process. Keratinocytes are cells that play a significant role in the process of wound healing and tissue regeneration. A human keratinocyte cell line (HaCaT) was exposed to an LFMF with low induction (180–195 Hz; 60 µT, magnetostimulation), red LED light (630 nm; 300 mW, LED therapy), and their combined action (magneto-LED therapy) in in vitro culture conditions. On day 4 and 8 of the experiment, the following parameters were determined: adhesion/proliferation, adenylate kinase (AK), nitric oxide (NO), cytokines (IL-1β, IL-6, IL-8, IL-10, IL-12p70, TNF-α), metalloproteinases (MMP-2, MMP-9), and collagen IV. It was shown that magnetostimulation caused an increase in keratinocyte adhesion/proliferation and IL-8 secretion and a decrease in IL-12 secretion. The LED therapy resulted in a transient increase in the secretion of NO and cytokines IL-1, IL-12, and IL-6 in keratinocytes. The use of magneto-LED therapy resulted in an increase in keratinocyte adhesion/proliferation, the secretion of cytokines IL-6 and IL-8, and NO with a simultaneous decrease in MMP-9 secretion. The results of our studies showed that the action of an LFMF with low-induction and LED light on keratinocytes can modulate the biological activity of keratinocytes towards improving the skin healing process.

## 1. Introduction

Keratinocytes are cells that, apart from creating a physical barrier protecting the skin against microorganisms, chemical compounds, and physical factors, play an important role in the healing process. This process consists of three, partially overlapping phases: (1) inflammation, (2) proliferation (so-called granulation tissue), and (3) remodelling [1]. The proliferative phase additionally includes three stages of repair: re-epithelialisation, fibroplasia, and angiogenesis. Viewing the skin healing process as a whole, we note that it includes cell migration to the site of damage, and cell proliferation and differentiation, as well as the synthesis, secretion, and remodelling of the extracellular matrix (ECM) [1]. In each of these phases, keratinocytes play a key part, influencing the rate of epidermal reconstruction and co-regulating the regeneration of deeper tissues. The inflammatory phase of the healing process is a tissue reaction, the aim of which is to isolate the damaged area, limit disturbances in the homeostasis of the damaged area, and prevent infection [2]. During this phase, keratinocytes synthesise and secrete a number of cytokines, including interleukins (e.g., IL-1, IL-6, IL-8, IL-12), tumour necrosis factor (TNF-α), and growth factors (e.g., TGF-α, TGF-β). These cytokines lead to the activation of neighbouring keratinocytes, fibroblasts, vascular endothelial cells, and lymphocytes, engaging them in the immune response and thus in the skin healing process. Interleukins secreted by keratinocytes participate, among others, in the membrane receptor activation of the capillary epithelium, increasing the permeability of blood vessels and facilitating the movement of immune cells towards the site of inflammation. They also promote the proliferation of fibroblasts and their synthesis of collagen and metalloproteinases (MMPs) in the later phases of healing [3]. Growth factor TGF-β secreted by keratinocytes is involved in the initial and final phases of healing. During the inflammatory phase, this factor demonstrates chemotactic activity towards leukocytes, recruiting them to the site of inflammation, while in the remodelling phase, it inhibits the angiogenesis process and regulates the production and remodelling of ECM by reducing the activity of fibroblast growth factors 1 and 2 (FGF-1, FGF-2) as well as epidermal growth factors (EGFs). TGF-β, together with TGF-β, also promote and control the migration, proliferation, and differentiation of keratinocytes, supporting re-epithelialisation and thus leading to a faster reconstruction of the epidermis and the formation of a protective barrier for granulation tissue on the surface [3]. Keratinocytes also synthesise and secrete a number of enzymes, including proteolytic ones involved in all phases of healing. These primarily include MMPs, mainly metalloproteinase 2 and 9 (MMP-2, MMP-9) [4]. In the healing process, the main task of MMP-2s is to degrade collagen types I, II, III, and IV, and also to promote cell migration and stimulate their apoptosis in the final phase of healing [5,6]. In turn, the task of MMP-9 is to degrade the basement membrane of blood vessels to the site of the inflammatory reaction. This occurs during processes related to the migration of leukocytes. MMP-9 also plays an important role in the remodelling of ECM components and in the degradation of chemokines as well as cytokines during the remodelling phase [5,6,7].

Keratinocytes themselves have receptors capable of binding to cytokines released by both resident skin cells and those migrating to the site of damage [2,8]. Keratinocytes express receptors for, i.e., TGF-β, TNF-α, and interferon γ (IFN-γ). These cytokines, acting on keratinocytes, inhibit their proliferation and stimulate cell migration into the temporary ECM scaffold of the skin [9]. Additionally, through keratinocytes, TNF-α leads to the secretion of pro-inflammatory interleukins (IL-1, IL-12), chemokines (CXCL8, CCL20), and adhesion-promoting molecules, e.g., ICAM-1 molecules [10]. Furthermore, TNF-α strongly interacts with other cytokines, e.g., IL-17, enhancing the immune response of keratinocytes [11]. In turn, IFN-γ is a well-characterised pro-inflammatory stimulus for keratinocytes because it strongly induces the expression of molecules important for the recruitment and activation of immunocompetent cells, including T lymphocytes [12].

The correct course of the healing process, including the proper functioning of keratinocytes, is often disrupted at various stages, which can result in the formation of chronic, difficult-to-heal wounds. The treatment of such wounds is still a serious medical problem. It is constantly prompting scientists to search for new therapeutic methods that support pharmacological approaches to treatment.

In recent years, special attention in this area of research has been paid to procedures in the field of physical medicine. One such procedure is magnetostimulation, which uses as a therapeutic factor alternating magnetic fields at low frequencies from several to 3000 Hz and with low magnetic induction between the range of 1 pT and 100 μT. They have a complex pulse shape and signal structure, providing a multi-peak frequency spectrum. The majority of therapeutic applications using an LFMF with low induction have proven to be effective [13,14,15,16]. For example, it has been shown that a series of magnetostimulation procedures accelerates the healing of venous leg ulcers, reduces the risk of their recurrence, and relieves swelling as well as pain in patients experiencing this condition [16,17].

Low-energy LED light therapy generated by Light Emitting Diodes (LEDs) in the visible red R (red) and near-infrared IR (infrared) ranges is gaining increasing interest in the context of regenerative medicine. LED lights of different wavelengths have a variety of effects on tissues, including regenerative processes such as wound healing. Red light, characterised by a wavelength within the range of approximately 620–750 nm, is of particular interest due to its potential pro-inflammatory and biostimulating effects. There are relatively few studies described in the literature on the effect of red LED light in healing chronic wounds. However, in the existing ones, a potential positive effect of this light is indicated on the healing process. The results of clinical studies allow us to confirm that this red LED light therapy (LED therapy) supports, among others, the healing process of diabetic ulcers, pressure sores, post-operative scars, and acne; inflammation reduction; and collagen synthesis stimulation [18,19,20,21,22]. For example, the team comprising Mancusi et al. (2024) demonstrated that the action of red light on wounds at a wavelength of 630 nm reduces the area of pathological changes and inflammation, and further ensures a reduction in bacterial load, thus shortening treatment duration and reducing amputation frequency [23].

The optimal therapeutic solution for accelerating the healing process of wounds, especially those difficult to heal, seems to be the simultaneous application of several methods. Due to the similar mechanism of action of magnetostimulation and LED therapy, attempts are being made to combine these two therapeutic methods in the treatment of chronic wounds, which gives rise to a new form of therapy called magneto-LED therapy, which can maximally support the healing process. The effectiveness of magneto-LED therapy in the treatment of lower limb venous ulcers was described in the latest study by Pasek et al. (2024), in which the authors showed that the use of combination therapy in patients leads to a significant reduction in the wound area, swelling, inflammation, and subjective feeling of pain [24].

Nonetheless, despite clinical studies indicating the efficacy of the described therapies in the treatment of problematic wounds, their action at a cellular level remains largely unknown. There is also a lack of studies regarding the possible synergistic effect of these therapies on skin cells. In order to assess the effect of magnetostimulation, LED therapy, and their combined action, and to provide further support for the therapeutic use of these methods in the healing of chronic wounds, the effect of magnetostimulation, LED therapy, and their combined action (magneto-LED therapy) on the biological activity of keratinocytes was assessed in an in vitro research model.

## 2. Results

**Figure 1:**
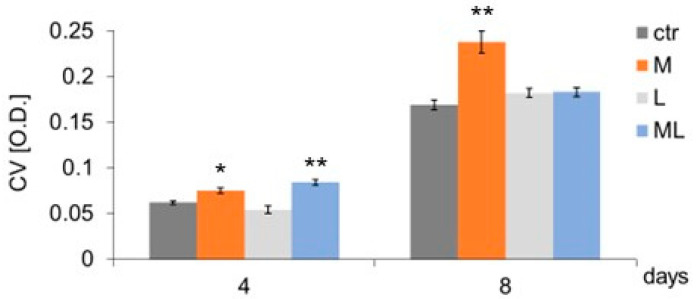
Effects of LFMF (magnetostimulation), red LED light (LED therapy), and their combined action (magneto-LED therapy) on keratinocytes’ adhesion of the HaCaT cell line. Cells were cultured for a specified number of days and were exposed 3 or 7 times to an LFMF with low induction (180–195 Hz; 60 µT, group M), red LED light (630 nm; 300 mW, LED, group L), or both simultaneously (group ML). On subsequent days of the experiment (4 or 8), cells were stained with crystal violet. O.D.—optical density was measured at 570 nm. Mean values ± SEM. *, **—differences between cells subject to the action of physical factors and cells not subject to the action of physical factors (group CTR) (* *p* < 0.05, ** *p* < 0.01).

**Figure 2:**
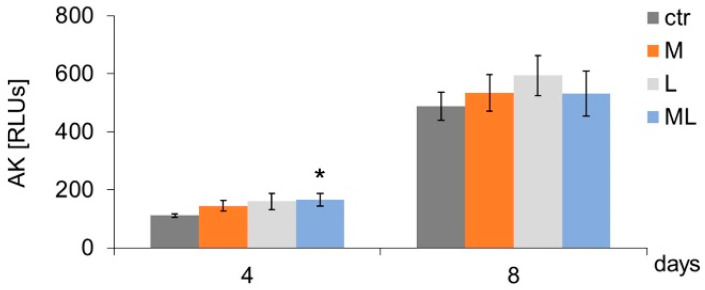
Effects of LFMF (magnetostimulation), red LED light (LED therapy), and their combined action (magneto-LED therapy) on cell adenylyl kinase (AK) release levels by keratinocytes of the HaCaT cell line. Cells were cultured for a specified number of days and were exposed 3 or 7 times to an LFMF with low induction (180–195 Hz; 60 µT, group M), red LED light (630 nm; 300 mW, LED, group L), or both simultaneously (group ML). AK levels were measured on the next 4 or 8 days of the experiment. RLUs—luminometer flux units. Mean values ± SEM. *—differences between cells subject to the action of physical factors and cells not subject to the action of physical factors (group CTR) (* *p* < 0.05).

It was shown that subjecting cells to three- and seven-fold magnetostimulation (4 and 8 days of the experiment, respectively) caused an increase in keratinocyte adhesion/proliferation (group M) compared to the control group (CTR) not subjected to magnetostimulation (Figure 1). Regardless of the number of LED light applications (three or seven) (group L), keratinocyte adhesion/proliferation did not differ from the adhesion/proliferation of cells in the CTR (Figure 1). An increase in cell adhesion/proliferation was found as a result of simultaneous, three-fold exposure to magnetostimulation and LED therapy on cells on day four of culture (ML group) compared to the CTR (Figure 1). Neither magnetostimulation (M group) nor LED therapy (L group), regardless of the number of applications, had any effect on the level of AK released from keratinocytes (Figure 2).

**Figure 3:**
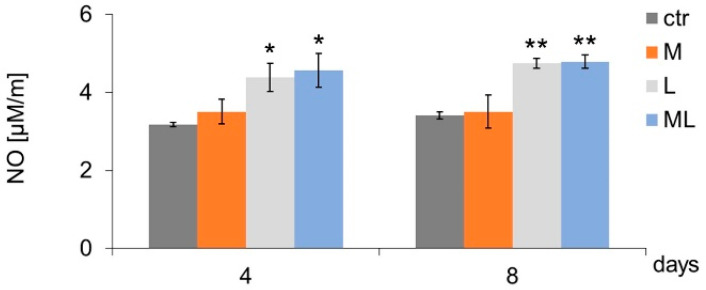
Effects of LFMF (magnetostimulation), red LED light (LED therapy), and their combined action (magneto-LED therapy) on the nitric oxide (NO) release levels by keratinocytes of the HaCaT cell line. Cells were cultured for a specified number of days and were exposed 3 or 7 times to an LFMF with low induction (180–195 Hz; 60 µT, group M), red LED light (630 nm; 300 mW, LED, group L), or both simultaneously (group ML). NO levels were measured on the next 4 or 8 days of the experiment. Mean values ± SEM. *, **—differences between cells subject to the action of physical factors and cells not subject to the action of physical factors (group CTR) (* *p* < 0.05, ** *p* < 0.01).

In the case of cells subjected to simultaneous (three-fold) exposure to magnetostimulation and LED light (ML group), a transient increase in the level of NO released from AK cells was observed (on day four of culture) in comparison to the CTR (Figure 2). Regardless of the number of magnetostimulation (M group) applications (three or seven), no differences were found in the level of NO secreted by cells in comparison to the CTR (Figure 3). Both three- and seven-fold exposures of cells to LED light (L group) and simultaneous exposure of cells to a magnetic field and LED light (ML group) induced an increase in NO secretion by keratinocytes, both on days four and eight of culture, compared to the CTR (Figure 3). 

**Figure 4:**
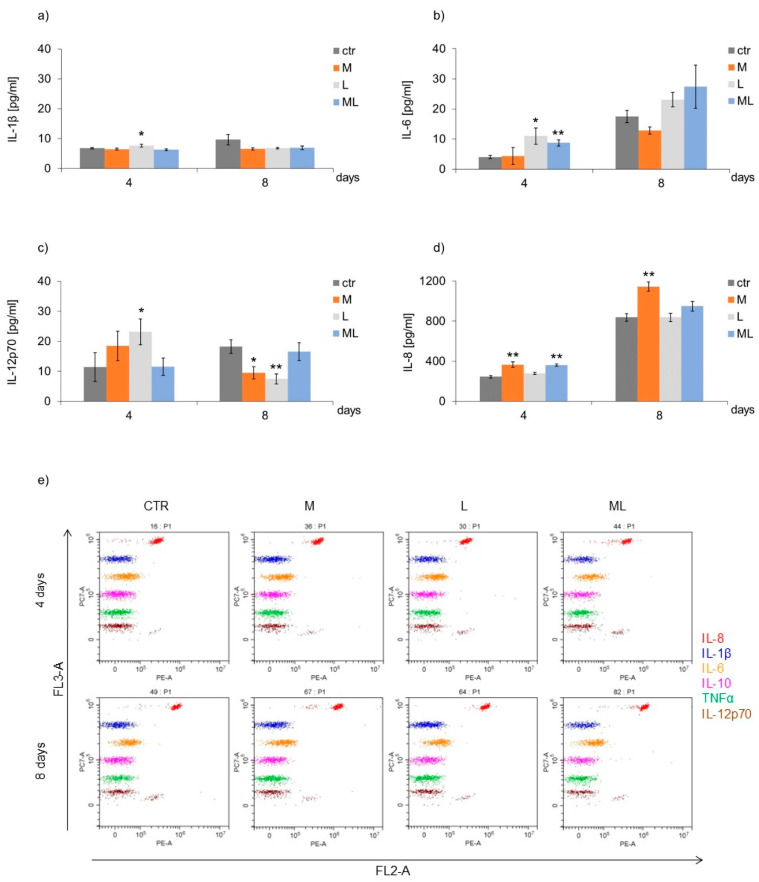
Effects of LFMF (magnetostimulation), red LED light (LED therapy), and their combined action (magneto-LED therapy) on the cytokines (**a**): IL-1β; (**b**): IL-6; (**c**): IL-12p70; (**d**): IL-8; secreted by keratinocytes of the HaCaT cell line, (**e**): example dot plots of the cytometric analysis of fluorescently labelled cytokines: IL-1, IL-6, IL-8, IL-12p70. Cells were cultured for a specified number of days and were exposed 3 or 7 times to an LFMF with low induction (180–195 Hz; 60 µT, group M), red LED light (630 nm; 300 mW, LED, group L), or both simultaneously (group ML). Cytokine levels were measured on the next 4 and 8 days of the experiment. Mean values ± SEM. *, **—differences between cells subject to the action of physical factors and cells not subject to the action of physical factors (group CTR) (* *p* < 0.05, ** *p* < 0.01).

Keratinocytes exposed to LED therapy three times (day four of culture, L group) secreted more IL-1-β, compared to the CTR (Figure 4a,e). In the case of cells exposed to LED light three times (L group) and to the simultaneous action of magnetostimulation and LED light (ML group), an increase in IL-6 secretion by the keratinocytes was observed on day four of culture, compared to CTR (Figure 4b,e). Keratinocytes exposed to LED light three times (L group) secreted more IL-12p70 compared to the CTR. 

In turn, exposing cells to seven-fold magnetic field stimulation (M group) and LED therapy (L group) caused the cells to secrete less IL-12p70 on the eighth day of culture compared to the CTR (Figure 4c,e). Cells exposed to three-fold magnetic field stimulation (M group) and simultaneous exposure to magnetic field stimulation and LED light (ML group) responded with an increase in IL-8 secretion on the fourth day of culture (Figure 4d,e). Longer, seven-fold (day eight of culture) exposure of the cells to magnetic field stimulation (M group) also resulted in an increase in IL-8 secretion compared to the cells from the CTR (Figure 4d,e). The presence of TNF-α and IL-10 cytokines was not demonstrated in any of the studied groups. 

**Figure 5:**
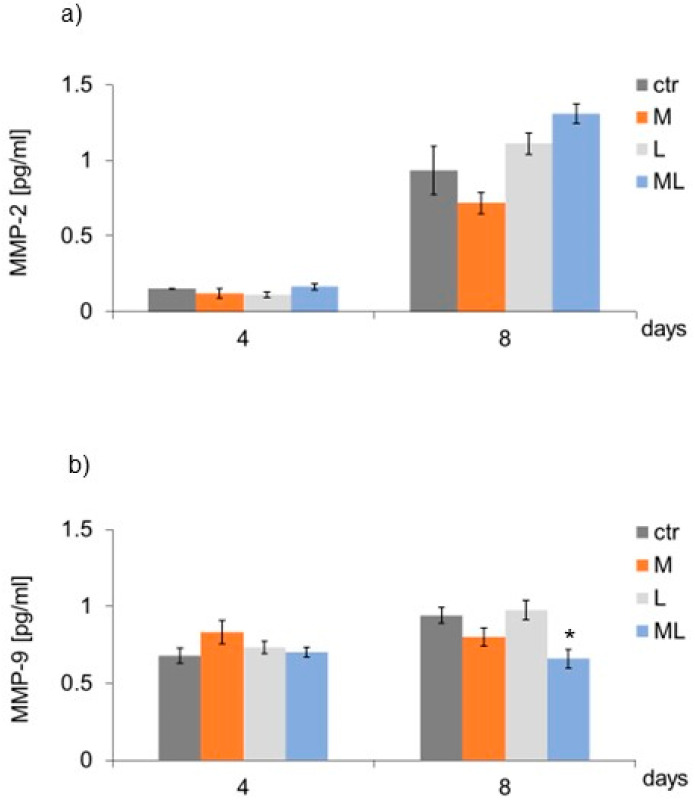
Effects of LFMF (magnetostimulation), red LED light (LED therapy), and their combined action (magneto-LED therapy) on (**a**) the level of metalloproteinases (MMP-2); and (**b**) the level of metalloproteinases (MMP-9), secreted by keratinocytes of the HaCaT cell line. Cells were cultured for a specified number of days and were exposed 3 or 7 times to an LFMF with low induction (180–195 Hz; 60 µT, group M), red LED light (630 nm; 300 mW, LED, group L), or both simultaneously (group ML). MMPs’ levels were measured on the next 4 and 8 days of the experiment. Mean values ± SEM. *—differences between cells subject to the action of physical factors and cells not subject to the action of physical factors (group CTR) (* *p* < 0.05).

**Figure 6:**
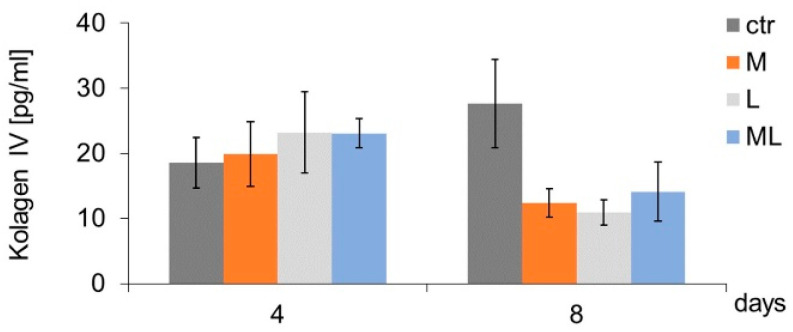
Effects of LFMF (magnetostimulation), red LED light (LED therapy), and their combined action (magneto-LED therapy) on the level of collagen (IV) secreted by keratinocytes of the HaCaT cell line. Cells were cultured for a specified number of days and were exposed 3 or 7 times to an LFMF with low induction (180–195 Hz; 60 µT, group M), red LED light (630 nm; 300 mW, LED, group L), or both simultaneously (group ML). Collagen IV levels were measured on the next 4 and 8 days of the experiment. Mean values ± SEM.

In the examined groups (M, L, and ML), no differences were found in the secretion of MMP-2 by keratinocytes, either on day four or eight of culture, compared to the cells representing the CTR (Figure 5a). In the case of MMP-9, a decrease in its secretion by keratinocytes was found on day eight of culture, under the influence of seven-fold exposure to simultaneous magnetostimulation and LED therapy (ML group), in comparison to the CTR (Figure 5b). On days four and eight of culture, in none of the examined groups of keratinocytes (M, L, or ML) were there any differences in the level of collagen IV secreted by the cells, as compared to the CTR (Figure 6).

## 3. Discussion

During the healing process, keratinocytes are activated. They proliferate and synthesise a number of active factors as well as ECM components in order to restore the integrity of damaged tissue [25]. It has been found that LFMFs affect various biological processes; however, knowledge on their effects and the way they occur is not fully understood. The results of the present study showed that exposure of keratinocytes to an LFMF with low induction can modulate the biological activity of keratinocytes. It has been demonstrated that magnetostimulation causes an increase in keratinocyte adhesion/proliferation, which is a beneficial phenomenon, because cell proliferation promotes the process of re-epithelialisation and the creation of a protective surface barrier for the formulation of granulation tissue. A rapid reconstruction of the epidermis, in turn, has positive influence on further stages of healing, reducing the probability of infections, maintaining homeostasis of tissue fluids, and supporting the reconstruction of the deeper dermis [26]. 

In the literature on the subject, there are few studies in which the effects of magnetostimulation on the biological activity of keratinocytes are assessed, including their adhesion/proliferation. However, those that do appear are consistent with the results of the current research. For example, Vinale et al. (2008) showed that keratinocytes exposed to a magnetic field for 72 h, at a frequency of 30 Hz and an intensity of 40 μT, exhibit an increase in proliferative activity [27]. Petruno et al. (2009) also showed an increase in proliferative activity of keratinocytes exposed to a magnetic field at a frequency of 50 Hz and an intensity of 1 mT for 3, 18, and 48 h [28].

The effectiveness of the healing process largely depends on balance between pro-inflammatory and pro-regenerative factors [29,30]. The microenvironment of a chronic wound is characterised by an excessive expression of pro-inflammatory mediators, such as TNF-α and IL-1β, IL-12, and MMPs produced by immunocompetent cells, but also, to a very large extent, by keratinocytes [9]. Data from the literature regarding the topic allow for the indication that these factors may have an inhibitory effect on the healing process. It has been shown, among others, that cytokines TNF-α and IL-1β present in a chronic wound inhibit the migration and proliferation of keratinocytes and fibroblasts. Additionally, such cytokines may induce apoptosis of these cells [31,32,33]. In turn, other researchers have demonstrated that the metalloproteinases, MMP-2 and MMP-9, present in chronic wounds can prolong the inflammatory phase of healing by delaying the onset of the proliferative phase in this process [34,35].

The results of our study allow us to indicate that magnetostimulation had no effect on the secretion of pro-inflammatory cytokines, i.e., IL-1, and TNF-α via keratinocytes, or resulted in a decrease in their secretion, as was the case with IL-12. The anti-inflammatory effect of a low-frequency (50 Hz) magnetic field was described by Patruno et al. (2018), who demonstrated a decrease in the secretion of IL-1β and TNF-α by keratinocytes 8 and 24 h following exposure to a magnetic field [28]. The results of the present study also suggest that magnetostimulation increased the secretion of chemokine 8 (IL-8) by keratinocytes. 

Data from the literature indicate that IL-8 is an important factor modulating tissue healing, as it is, among others, a significant chemoattractant for keratinocyte migration and proliferation [36,37]. The team comprising Jiang et al. (2012) demonstrated that IL-8 and the expression of IL-8 receptors (IL-8Rs) are useful indicators regarding the nature of wound healing, indicating that in chronic wounds, IL-8 receptor levels (IL-8R) are significantly lower compared to acute wound tissues. The published research results indicate that a loss or reduction in IL-8R may be an important part of the mechanisms responsible for the lack of wound healing and, at the same time, evidence of the significant role played by IL-8 in modulating the healing process and preventing the formation of chronic wounds [37]. In reference to these studies, the results obtained in the experiments conducted in this research confirm that subjecting keratinocytes to magnetostimulation promotes their activation in a direction supporting the processes occurring in the proliferative phase of wound healing. 

In the published in vitro models, it has also been shown that LED therapy can improve the wound healing process. However, well-defined parameters of different light sources for this therapy are still insufficient. The lack of in vitro studies on determining the biological activity of keratinocytes exposed to red LED light is complemented by the experiments conducted in this trial. The results of this research indicate that exposing keratinocytes to LED light can modulate their biological activity, among others, by increasing the secretion of nitric oxide by cells. NO is involved in many physiological cellular processes, including those that are pathological. It has been shown that NO, depending on its concentration, can have both pro- and anti-apoptotic effects on different types of cells affected by it [38]. In high concentrations, NO, reacting with proteins, can cause the nitrosylation of amine residues, impairing their function and causing a cytotoxic/apoptotic effect on the surrounding cells. It can also stimulate the secretion of pro-inflammatory cytokines, prompting cells of the immune system and initiating inflammation. However, increased NO levels are not a clearly negative phenomenon. There are scientific reports in which it has been noted that increased NO levels accelerate the proliferation of fibroblasts and keratinocytes, as well as collagen synthesis, contributing to faster wound healing [39]. According to the research carried out by Hourelda et al. (2010), NO levels are significantly reduced in difficult-to-heal diabetic wounds, and their elevation may accelerate the healing of these lesions [40]. According to Han et al. (2012), NO concentration levels increase rapidly after skin damage and then gradually decrease as the wound healing process progresses. It has also been shown that elevated NO levels accelerate fibroblast proliferation [41]. In turn, Witte et al. (2000) noted that there is a correlation between induced, elevated NO synthesis in the wound and increased collagen production. In their research, the authors also observed an increase in collagen production by normal/undamaged fibroblasts induced by NO [42]. Due to the fact that a higher level of NO may project into an increase in the process of collagen fibre formation, it seems that the rise we observed in the level of NO secreted by the keratinocytes exposed to the LED light may be a beneficial phenomenon.

The results of the current research made it possible to suggest that the increase in NO secretion by irradiated keratinocytes was accompanied by an increase in the secretion of pro-inflammatory cytokines IL-1 and IL-12, as well as IL-6, which is characterised by the dual nature of both pro- and anti-inflammatory action. It should be emphasised, however, that the increase in the secretion of these cytokines was transient, because a larger number of cell irradiation applications had no effect, and in the case of IL-12, it caused a decrease in the secretion of this cytokine. The results of this study indicate that the type of applied LED therapy has an effect on the increase in the secretory activity of keratinocytes towards the acceleration of the phases related to inflammatory processes.

Combination therapies enable the simultaneous action of several physical factors on biological processes occurring in cells, which may increase the effectiveness of each of the implemented methods. Because the healing processes begin before the development of a full inflammatory response, understanding how resting cells respond to physical factors helps to assess whether a given therapy will be able to support tissue regeneration and reconstruction in conditions without developed inflammation and whether the applied values of physical factor parameters will not additionally intensify the inflammatory reaction.

The results of the present study have exhibited that the simultaneous use of magnetostimulation and LED therapy had an effect on modulating the biological activity of keratinocytes, as it resulted in increased cell adhesion/proliferation, increased NO secretion, and a transient increase in the secretion of IL-6 and IL-8 cytokines and a decrease in the secretion of MMP-9. The demonstrated increased adhesion of keratinocytes is crucial for the reconstruction of the epidermis. On the other hand, increased NO secretion may stimulate angiogenesis and modulate inflammatory responses. At the same time, although cytokines are associated with the intensification of the inflammatory response, their short-term increase is beneficial because it mobilises immune cells to the site of damage, accelerating the initiation of repair processes. Excess MMP-9 can delay healing through excessive tissue degradation. Its reduced secretion indicates the stabilisation of the repair process, which supports the healing and restoration of the skin structure. The results of our study are a reference point and a kind of standard for the results of subsequent experiments. Knowing the type and strength of the response of keratinocytes in a normal state to the described physical factors, it will be possible to more precisely assess the cellular response in an inflammatory state and refine the size of the applied values of the physical factor parameters, and thus obtain an answer to the question of whether the factors we are evaluating can be significantly useful in the process of tissue healing. 

## 4. Materials and Methods

### 4.1. Keratinocyte Culture Conditions

Research was conducted on the human keratinocyte HaCaT cell line that was purchased from the American Type Culture Collection (CLS Cell Lines Service, Eppelheim, Germany), which was cultured in vitro. The cells were cultured in 75 mL culture flasks (Googlab Scientific, Charlottesville, VA, USA. LLC) in a DMEM culture medium (Lonza, Bend, OR, USA), with the addition of 10% bovine serum (FBS) (Thermo Fisher, Gibco, Waltham, MA, USA 02451 USA) and a 1% solution of antibiotics, penicillin and streptomycin (Thermo Fisher, Gibco, Waltham, MA, USA 02451), as well as a 2% L-glutamine solution (Biowest, Bradenton, FL 34211, USA), in an atmosphere of 5% CO_2_ and at a temperature of 37 °C. Cells from passages four–five were used for the experiment. The cell suspension for the experiment was obtained by adding the Triple to the culture flask (Lonza, Bend, OR, USA). After centrifugation (HERMLE Labortechnik GmbH, Wehingen, Germany), the cells were adjusted to a concentration of 0.01 million cells/mL, after which 1 mL of the cell suspension was placed in the wells of a 24-well culture plate (Nest SB, Woodbridge, NJ, USA). Cells were cultured in 24-well plates, in a HeraCell incubator (Heraeus, Thermo Scientific, Bremen, Germany), for four and eight days, respectively.

During the experiment, keratinocytes in a normal state were exposed to an LFMF (Viofor JSP Clinic device, Shenzhen, China) and red LED light (Viofor JPS system Magnetic Light device) or both simultaneously for 24 min, every 24 h, three or seven times using the following parameters:

- M group—LFMF with a frequency of 180–195 Hz and an induction value of 60 µT—magnetostimulation (M);
- L group—red LED light with a power of 300 mW and a wavelength of 630 nm—LED therapy (L);
- ML group—the combined action of an LFMF with a frequency of 180–195 Hz and an induction of 60 µT and red LED light with a power of 300 mW and a wavelength of 630 nm—magneto-LED therapy (ML);
- CTR—control cells not subject to the action of physical factors.

On the fourth (three-fold exposure of cells to a physical factor) and on the eighth days (seven-fold exposure of cells to a physical factor) of the experiment, the following parameters were determined: cell adhesion/proliferation (CV test), the level of adenylate kinase (AK; ToxiLight test), the level of nitric oxide (NO) (Griess test), secreted cytokines (CBA kit), and secreted metalloproteinases MMP-2 and MMP-9, as well as the level of secreted collagen IV. The level of collagen and MMPs was determined via the immunoenzymatic method (ELISA test), using commercially available sets of reagents characterised by appropriate sensitivity and specificity.

### 4.2. Cell Adhesion/Proliferation (CV Test)

The cell adhesion ability was examined using the crystal violet uptake test. The cells attached to the substrate were fixed with 2% paraformaldehyde—PFA (Sigma-aldrich, St. Louis, MO 63178, USA) for five minutes, stained for five minutes with the 0.5% crystal violet solution (Sigma, USA), and rinsed with water. Then, the dye absorbed by the macrophages was extracted by adding 0.5 mL of 100% methanol (Linegal Chemicals, Warszawa-Bemowo, Poland) to each well. The optical density (OD) of the fluid was read using the FLUOstar Omega reader (BMG Labtech, Ortenberg, Germany) at a 570 nm wavelength.

### 4.3. Level of Released Adenylate Kinase (AK) (ToxiLight Test)

The AK level was determined via quantification, using the bioluminescence method (Toxilight, Lonza, Switzerland). The supernatant from the cell culture (20 µL) was collected and transferred to a white, 96-well plate (Nest SB, Woodbridge, NJ, USA). Then, 100 µL of the AK Detection Reagent solution was added to each well, according to the manufacturer’s protocol. After five minutes of incubation, the luminescence value was read using the FLUOstar Omega reader (BMG Labtech, Ortenberg, Germany).

### 4.4. Level of NO Secretion (Griess Test)

The secreted nitrite ion amount was determined according to the following procedure: In total, 100 μL of the cell supernatant was transferred to each well of a 96-well plate (Nest SB, Woodbridge, NJ, USA). Then, 100 μL of a 1:1 reagent mixture was added (Sigma-Aldrich, Germany)—Griess A (1% sulphanilamide in 5% phosphate acid) and B (0.1% (1-naphthyl) ethylenediamine in H_2_O). After five minutes, the optical density (OD) of the liquid was read at a wavelength of 540 nm, using the FLUOstar Omega reader (BMG Labtech, Ortenberg, Germany).

### 4.5. Level of Secreted Cytokines (Analysis of Flow Cytometry)

Cytokine levels in the cell culture supernatants were measured via flow cytometry using Flex Set kits (CBA, BD Biosciences, San Diego, CA 92126, USA). The entire assay procedure and all measurements and analyses were performed in accordance with the manufacturer’s instructions, using the Beckman Coulter flow cytometer (Life Science, Indianapolis, IN, 46268, USA). Using the Mouse Inflammation Kit (BD Biosciences, San Diego, CA 92126, USA), the simultaneous determination of six cytokines was possible: interleukin (IL-1β), interleukin 6 (IL-6), interleukin 8 (IL-8), interleukin 10 (IL-10), interleukin 12p70 (IL-12p70), and tumour necrosis factor (TNF-α). The data analyses and determination of cytokine concentrations were performed in Microsoft Excel, using standard curves based on subsequent dilutions of the standard.

### 4.6. Level of Secreted MMP (Elisa Test)

The levels of secreted MMP-2 and MMP-9 were determined in the plasma via an enzyme-linked immunosorbent assay (ELISA). The analyses were performed according to the manufacturer’s instructions (Cloud-Clone Corp. Houston, TX, USA).

### 4.7. Level of Secreted Collagen IV (Elisa Test)

The level of secreted collagen IV was determined in the plasma by an enzyme-linked immunosorbent assay (ELISA). The analyses were carried out according to the manufacturer’s instructions (Cloud-Clone Corp. Houston, TX, USA).

### 4.8. Statistical Analysis

The data are presented as mean values and standard errors. The Shapiro–Wilk test was conducted to assess data for normality of distribution. A one-way analysis of variance (ANOVA) was employed for the evaluation of significances in the differences in the measurement variables across study groups. When a significant main effect was detected, Tukey’s post hoc test was introduced. Differences were considered statistically significant if the level of test probability was lower than the assumed level of statistical significance (*p* < 0.05).

## Data Availability

The raw data supporting the conclusions of this article will be made available by the authors on request.
